# Intraocular Tumors in Horses: Diagnosis, Tumor Classification, Oncologic Assessment and Therapy

**DOI:** 10.3390/vetsci12101006

**Published:** 2025-10-17

**Authors:** Christopher Ostendarp, Ann Kristin Barton

**Affiliations:** 1 Equine Clinic Hochmoor, Ruthmannstr. 10, 48712 Gescher, Germany; 2 Veterinary Department, Freie Universitaet Berlin, Oertzenweg 19b, 14163 Berlin, Germany

**Keywords:** tumor, horse, intraocular, malignant, equine oncology, ophthalmic imaging, tumor classification

## Abstract

**Simple Summary:**

Intraocular malignant neoplasia is a rare condition in horses and only single case reports exist. This review contains a systematic overview of the current literature and aims to provide a clinicopathologic classification of the currently described intraocular neoplasia. This seems important for any consideration concerning therapy and prognosis. Diagnosis of intraocular neoplasia can be achieved with clinical ophthalmic examination, supported by basic medical imaging techniques. A systematic oncologic workup and tumor staging seem to be rarely undertaken in equine ophthalmology. This review emphasizes the importance of these aspects and demonstrates the current lack of therapeutic options in equine ophthalmology as well as the comparative oncology perspective. The developments in human and small animal medicine seem to be promising, although some limitations remain unsolved.

**Abstract:**

Intraocular neoplasia in horses is rare and only few case reports and small case series exist. Intraocular neoplasia has various clinical signs and includes important differential diagnoses in ocular disease. This narrative review of the current literature aims to provide a clinically relevant overview and classification of intraocular tumors in horses and adds a comparative oncological perspective concerning diagnosis, treatment and future considerations. The available clinical and imaging examination techniques allow for a reliable and differentiated investigation of the tumor, even in the standing horse, using high-frequency ultrasound or optical coherence tomography, which have gained importance in equine ophthalmology. Sectional imaging techniques, in particular computed tomography, are suitable for the examination of the peribulbar, retrobulbar and orbital structures. Differentiated diagnostics including precise tumor staging (TNM: tumor, node, metastasis) are essential for a general prognostic and therapeutic assessment. The embryologic and anatomic tissue origin of the neoplasm is the basis for clinicopathologic classification. Medulloepithelioma and uveal melanocytic neoplasia are the most common intraocular tissue formations occurring in horses. Whereas melanocytic neoplasia of the iris can be treated surgically, neuroepithelial tumors regularly lead to bulbus extirpation. Other primary intraocular neoplasms are sporadically reported, as well as intraocular metastasis of systemic neoplasia. Chemotherapy and radiation therapy are not currently used to treat intraocular neoplasia in horses and need to be further investigated, especially regarding the latest developments in human and small animal medicine. In addition, horses and dogs may serve as models for human oncologic research.

## 1. Introduction and Methodology

Intraocular tumors are a rare condition in horses and only single case reports exist. Due to their local growth and tissue invasion, every intraocular neoplasia fulfills the criteria for malignancy sooner or later. Broad-based histopathological studies concerning tumor types and categorization are lacking in equine ophthalmology, although this is fundamental for the development of differentiated diagnostic and therapeutic approaches.

This narrative review provides a structured overview of the current literature on clinical diagnostic opportunities including advanced imaging opportunities in equine ophthalmology. Due to the current literature base lacking standardized retrospective or prospective case studies on intraocular tumors in horses, a systematic review was impossible at this time. Nevertheless, this review aims to provide ideas for developing standardized diagnostic and therapeutic protocols and thus provides a basis for systematic studies on intraocular neoplasms in horses. Based on models for classification of neoplasia in human and veterinary medicine [1,2], a clinicopathologic classification of currently described intraocular neoplasia in the horse is provided.

In human medicine, a higher incidence allows for a better review of cases and the development of therapeutic options [3,4,5]. An interdisciplinary work-up is the standard approach in human medicine [3,6], but is rarely reported in equine ophthalmology. Based on the current oncologic procedures in equine medicine, possible diagnostic steps for an oncologic work-up will be summarized. In human oncology, intraocular metastases of malignant cancer are ten times more common than primary intraocular tumors [3,6]. The effects of systemic neoplasia on the equine eye have not been systematically studied.

This lack of information leads to limited therapeutic approaches for (malignant) intraocular tumors in horses. An interdisciplinary and interspecies summary of therapeutic options in human and small animal ophthalmic oncology can provide a basic idea for the development of multimodal, interdisciplinary approaches for diagnosis and treatment.

## 2. Basic Oncologic Theory for Intraocular Neoplasia in Horses

Intraocular neoplastic disease is a rare condition in equine patients and may be considered too rarely a differential diagnosis. Not only to assess the possible outcome for the affected eye, but also because of the systemic effects of neoplastic disease, a fundamental understanding of oncologic basics needs to be fundamental knowledge for equine ophthalmologists too.

Depending on the type and location of the neoplasm, the diagnosis and treatment can be challenging [7,8,9], and for the assessment of intraocular neoplasms, a differentiated view on internal neoplasms and their clinical signs is necessary [7,10]. External neoplasia have a higher incidence in horses, mostly present with more obvious clinical signs and are more accessible using diagnostic approaches, also for those surrounding the eye [7,11]. Unfortunately, this differentiation is difficult concerning intraocular tumors. Although classified as an external periocular neoplasm, cases with intraocular effects due to their peribulbar or retrobulbar growth [12,13] or bulbar and intraocular tissue invasion [14,15,16,17] are reported.

Division of benign and malignant tumors, based on general oncologic knowledge [7,18,19] and pathologic classification [1,20,21], can only be the first step in viewing intraocular neoplasms. Due to intraocular growth, local tissue infiltration and the resulting functional impairment of the globe, many intraocular neoplasms can fulfill the criteria for malignancy [22,23,24,25,26,27,28,29] but may be diagnosed with benign histopathological criteria [11,19,20,21,30,31]. For the clinician, this must be carefully taken into consideration when assessing individual cases to decide on therapy and prognosis.

It is necessary to understand the molecular dimension of tumorigenesis [19,31,32]. Exogenous or endogenous factors (carcinogens) cause non-lethal DNA damage to a cell [31]. If this results in failure of the DNA repair mechanisms, multiple mutations occur in the genome of the somatic cells [31]. This includes the activation of growth-promoting oncogenes, the inactivation of apoptosis genes and the inactivation of tumor suppressor and DNA repair genes [31]. This leads to the expression and synthesis of altered gene products with the subsequent loss of regulatory gene products, which promotes clonal expansion, further mutations and increased cellular heterogeneity, ultimately resulting in the development of a (malignant) tumor [31]. Another factor in tumorigenesis is the “tumor microenvironment”, which contains multiple cellular and non-cellular components that help tumors to acquire their biological characteristics [33]. Furthermore, tumor interaction with the hosts’ conditions (e.g., inflammation) plays a particular role in tumorigenesis [34,35]. The individual molecular approach to cancer for differentiated and even individual diagnostic and targeted therapeutic access became a key tool in human oncology and remains a great opportunity for veterinary cancer research [19]. No differentiated research on the molecular characteristics of equine intraocular tumors and their possible application for diagnostic and therapeutic purposes has been published so far. Nevertheless, intraocular tumors (e.g., uveal melanoma) already play a role as a possible model for comparative oncological studies (see comparative oncology: perspective considerations). In general, the immune privilege of the eye and resulting possible immune escape mechanisms can be assumed to be a factor favoring intraocular tumorigenesis, especially the inner eye, which has already been proven in human medicine [36,37].

The differentiation between a primary intraocular neoplasm and a metastasis of systemic neoplastic disease (e.g., lymphoma, adenocarcinoma) [8,38,39,40,41,42,43] is important. It is also important to recognize that there will be cases of intraocular neoplasms where this distinction cannot be made definitively [44,45,46], and this can be explained in particular by the limitations of diagnostic options. Nevertheless, these cases require differentiated considerations regarding the therapeutic and prognostic approach.

## 3. Diagnosis

The eye is an organ that is easily accessible for diagnostic purposes. Neoplasia can be visualized and assessed via clinical examination and medical imaging techniques. Even the peribulbar, retrobulbar and orbital structures can be examined by using different diagnostic tools and a complete ophthalmic diagnostic approach in cases of suspected ocular neoplasia. As described above, intraocular neoplasia can be assigned to the group of internal neoplasms [7,47] or can even be a result of them, which makes the diagnostic approach even more difficult because the clinical signs of internal neoplasia are mostly unspecific [7,8,39,40,47].

The general oncological assessment of an equine patient does not follow a clear structure, often is not part of the clinical routine and may be underestimated in the work-up of an equine ophthalmic patient. This is why this review aims to provide a summary of opportunities for a systematic, structured oncologic work-up in cases of intraocular tumors that can complement the ophthalmic diagnostic approach.

### 3.1. Clinical Ophthalmic Examination

As mentioned above, almost all intraocular neoplasms result in clinical ophthalmic signs [10,22,23,24,48,49] that can have an inflammatory, degenerative and functional nature, which may temporarily, permanently or irreversibly impair vision, resulting in the patient being presented at the ophthalmology practice.

Querying the medical and ocular history of the horse is followed by a general clinical examination and is the basis of assessing the patient [50].

The ophthalmic exam is the most direct way to visualize a tumor and evaluate its effects on the organ. It must include in a routine manner an examination of both eyes, containing the steps described elsewhere [50]. For intraocular neoplasia, possible clinical signs are summarized in Table 1.

**Figure 1:**
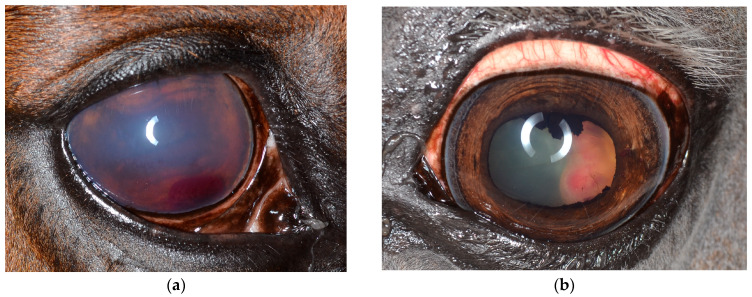
Clinical ophthalmic findings in the case of intraocular neoplasia in the posterior segment: (**a**) Severe acute uveitis in the case of an undifferentiated malignant tumor of the ciliary body due to corneal opacification, dense flare and hyphema affecting the anterior chamber and the miotic pupil; the neoplasia is not accessible for clinical ophthalmic examination. (**b**) A case of a medulloepithelioma of the posterior segment presented without ocular pain, positive light reflexes, mild conjunctival hyperemia and signs of previous uveitis (posterior synechiae).

**Figure 2:**
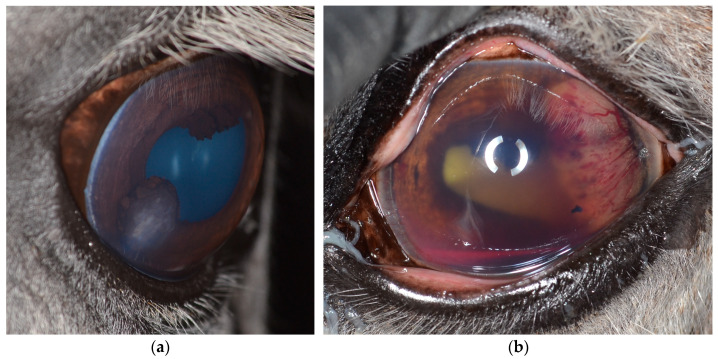
Clinical ophthalmic findings in a case of intraocular neoplasia affecting predominantly the anterior segment: (**a**) a case of an iris melanoma touching the corneal endothelium which leads to circumscribed endothelial opacification, mild stromal edema and focal neovascularisation; (**b**) a medulloepithelioma affecting large parts of the iris and iridocorneal angle and leading to signs of acute uveitis (hyphema, flare, free pigmented precipitates).

The most common reasons for presentation are impairment of vision or blindness [13,25,26,27,28,48,51,52,53,54,55]. Obvious symptoms, such as exophthalmos or ocular pain, are frequently reported, but may not be present, especially in medulloepithelioma (Figure 1b) [25,52,62] or intraocular melanoma (Figure 2a) [29,59,60]. The predominant finding of the inner eye is tissue formation, especially in the anterior segment affecting the iris and iridocorneal angle (Figure 2) [26,29,38,43,48,52,53,56,57,58,59,60]. Additionally, signs of uveitis (Figure 1a and Figure 2b; Table 1: conjunctival/scleral hyperemia, corneal edema and neovascularization, miosis, flare, hyphema, hypopyon, iris abnormalities) and resulting glaucoma [26,27,29,48,53] are found frequently.

Table 1 highlights the lack of specificity and wide variability of clinical signs in intraocular neoplasms. It is therefore important to emphasize that neoplastic changes must always be added to the list of differential diagnoses for examining ophthalmic patients and that a precise etiological assessment to determine whether disease origin is primary inflammatory, degenerative or even neoplastic is essential.

### 3.2. Imaging Techniques

Ultrasonographic examination of the eye and peribulbar structures is widely used and makes the investigation of intraocular tumors accessible for routine practice [63,64,65,66,67,68]. Under hospital conditions, sectional imaging techniques, especially computed tomography and standing low-field magnetic resonance imaging, are commonly available [64,68,69,70]. To assess intraocular neoplasia, high-field magnetic resonance imaging (MRI) of the anesthetized horse is the gold standard, but is not commonly available in equine clinics apart from teaching hospitals [13,68,69]. However, high-frequency ophthalmic ultrasound and ocular coherence tomography or even in vivo confocal microscopy can compete with MRI and even be performed easily in the standing horse [67,71,72]. Skull radiography is rarely reported in cases of intraocular neoplasia [64,67], but can be helpful, when periorbital invasion with orbital involvement is questionable and computed tomography is not available.

Imaging plays a particularly important role in tumor staging, as it is important not only for searching for potential metastases, but also for examining regional and distant lymph nodes [7,73]. Since only the mandibular lymph nodes are palpable in the physiological state, imaging of other clinically relevant lymph nodes (medial retropharyngeal, cranial and caudal deep cervical, caecal, iliosacral lymphocentre) is required, for which initial reference studies on ultrasonographic examination in horses are available [73]. Possible pathological conditions must be investigated in future studies, whereby assumptions from basic propaedeutics should be taken as the basis for clinical considerations until then.

#### 3.2.1. Ultrasonography

**Figure 3:**
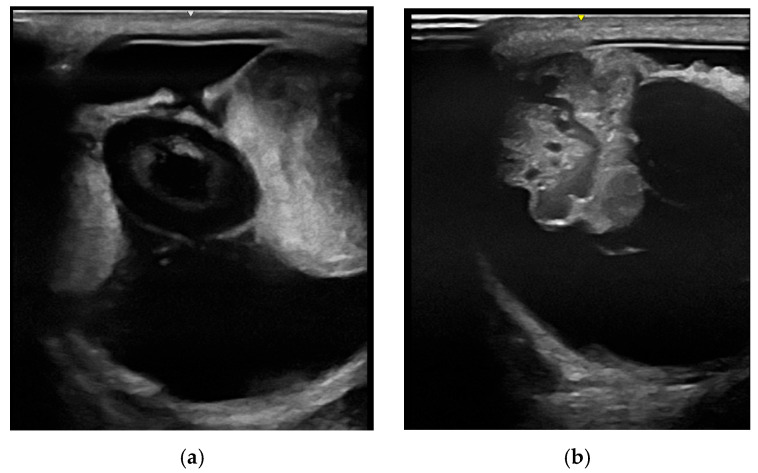
The use of diagnostic (transpalpebral) B-mode ultrasound investigating intraocular neoplasms: (**a**) due to the miotic pupil, tissue formation in the posterior eye and even the subluxated lens could not be investigated via clinical ophthalmic exam, but could be easily visualized by sonography; (**b**) the ultrasonographic appearance of a medulloepithelioma originating from the ciliary body.

B-mode ultrasound (Figure 3) using frequencies between 5 and 15 MHz is commonly used in equine practice to examine the eye as an addition to the clinical ophthalmic exam, especially in cases of a miotic pupil or opacifications of the anterior segment, to assess the posterior eye segments [63,64,65]. To achieve the best possible imaging, it is essential to understand the technical possibilities and select the correct settings, including those concerning the transducer, frequency and focus position. However, in general, diagnostic ultrasound is relatively easy to perform and enables the practitioner to achieve reliable examination results [67,74]. Although this cannot compete with high-frequency ultrasound, minor structural deviations (e.g., Descemet membrane detachments) can also be visualized this way [67,74,75,76].

**Figure 4:**
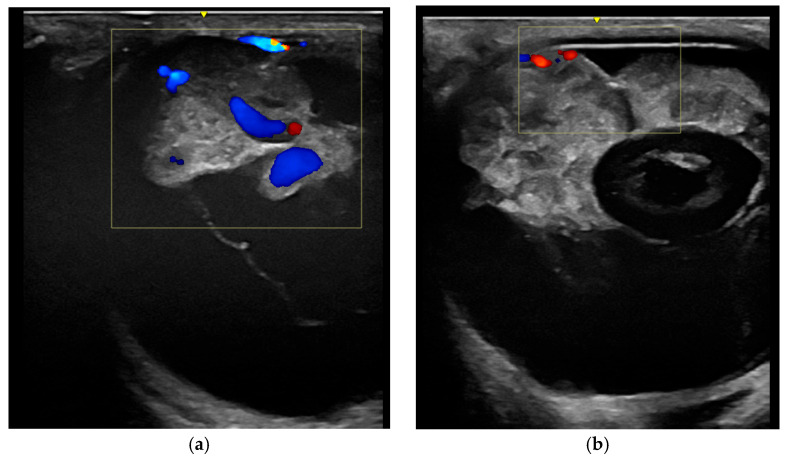
Comparison of the Doppler signal to visualize vascularization: (**a**) medulloepithelioma; (**b**) undifferentiated malignant tumor.

Doppler ultrasonography (Figure 4) is a standard feature of modern ultrasound machines and is important for investigating the grade and structure of blood supply of intraocular tissue formations [26,64,74,76]. This may help with tumor classification and provides important information for the planning of possible therapeutic and surgical procedures.

**Figure 5:**
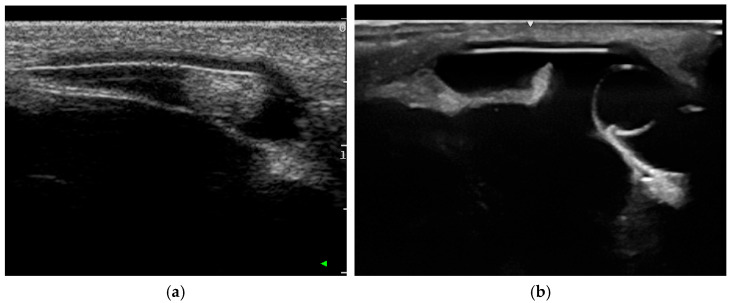
The use of diagnostic B-mode ultrasound (transpalpebral, anterior segment) in differential diagnostics: (**a**) iris melanoma with continuous solid echotexture touching the corneal endothelium; (**b**) iris cyst with an anechogenic lumen.

Ultrasound is important in differential diagnostics (Figure 5). In particular, iris cysts must be clearly differentiated from iris melanoma or melanocytoma, which is easily possible with ultrasound [64,65,74].

Beyond this commonly available ultrasound technique, high-frequency ultrasound (frequencies from 20 to 38 Mhz) and ultrasound biomicroscopy (frequencies from 40 to 100 Mhz) are gaining importance in equine ophthalmology [64,67,75]. By using these, a detailed and near-microscopic resolution is achieved and allows for a differentiated visualization of the anterior segment that is close to the transducer [64,75]. Until now, this has predominantly been used to assess corneal changes and structural deviations of the iridocorneal angle [67,77], but it is promising for the visualization of intraocular masses too as a tool for planning surgical access. Further studies are needed to assess the possibilities and limitations of these procedures in cases of intraocular neoplasia.

#### 3.2.2. Sectional Imaging

**Figure 6:**
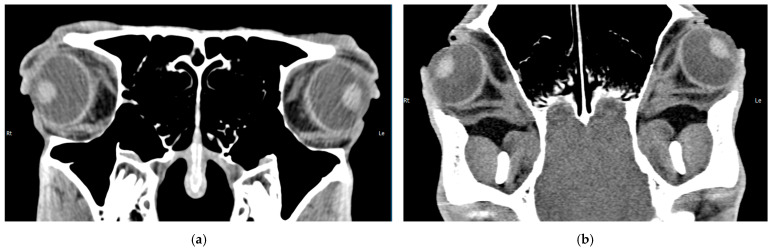
A CT scan of a horse without ophthalmic disease; the retrobulbar structures can be visualized by using soft tissue settings: (**a**) craniocaudal view; (**b**) dorsoventral view.

As mentioned above, computed tomography (CT) is commonly available and used in equine medicine to examine the head, and it is possible to perform it on a standing horse under sedation [67,69,70,72,76]. Its technical features enable differentiated visualization of soft tissue (Figure 6). In particular, the peribulbar and retrobulbar area can be examined, where ultrasound reaches its limits [67,71,74,75].

**Figure 7:**
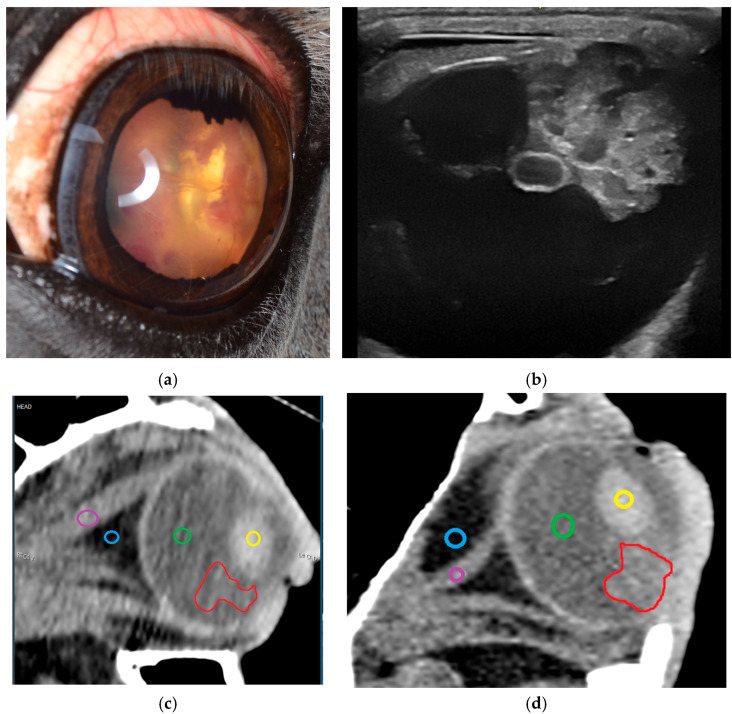
A digital photograph (**a**) and the ultrasonographic appearance (**b**) of an intraocular medulloepithelioma compared to a CT scan (**c**,**d**) in a horse with medulloepithelioma (framed in red) affecting the posterior segment originating from the ciliary body: (**c**) caudocranial view; (**d**) dorsoventral view. (**c**,**d**) The attenuation coefficients are measured in universal Hounsfield Units (HU). Intraocular tissue formation/medulloepithelioma (red): 61–87 HU; lens (yellow): 101–172 HU; vitreus (green): 19–34 HU; retrobulbar fat (blue): −110 HU; retrobulbar soft tissue/muscles (purple): 50–80 HU.

Figure 7 visualizes the limited usability of computed tomography for intraocular neoplasia and compares it to access via clinical and ultrasonographical examination. Although easy to perform even in the standing horse [67,69,70,72] and despite the possibility of visualizing the neoplasia with measurable attenuation coefficients (Figure 7c,d), transpupillary visualization (Figure 7a) and the ultrasonographic appearance (Figure 7b) allow for a much more detailed examination. Particularly in cases where there is no concurrent inflammatory disease and where diagnostic mydriasis is possible, clinical examination, including post-pupillary examination, yields good results in the assessment of intraocular neoplasms (Figure 7a). Sonography completes this examination with high-resolution images of the inner eye (Figure 7b). Figure 7b,c show the computed tomography image of the tumor from Figure 7a,b: although the tumor can be detected on the image and can also be differentiated from the surrounding structures by measurement of attenuation coefficients, the level of detail cannot compete with clinical and ultrasonographic examination.

However, as cases of intraocular tumors invading the peribulbar and retrobulbar space have been described [13,25,26,27], a CT scan should be considered if there is uncertainty about this eventuality.

#### 3.2.3. Optical Coherence Tomography (OCT) and Other Advanced Imaging Techniques

Spectral domain OCT (SD-OCT) has become important in understanding ocular and, in particular, corneal disease in horses over the last few years, as it can be performed in the standing sedated horse [67]. OCT provides high-resolution sectional images via interferometry, which correlates light scattered back from tissue and light that travels a known distance through tissue [78,79]. This physical principle limits the use of OCT, as it needs clear media to enable penetration of the light beam. Furthermore, the patient’s cooperation is an important criterion, as the procedure is susceptible to motion artefacts [78]. SD-OCT has already been studied in horses for corneal disease [80] and for the evaluation of the retina and optic nerve [78]. The potential benefits for the diagnosis of intraocular neoplasia seem promising because of the high resolution compared to sonography and other sectional imaging methods [78,80], but further studies are essential.

While OCT and other advanced techniques such as specular microscopy or in vivo confocal microscopy are currently used to study anatomy and describe more common diseases [64,67,75,78,79,80], these techniques must also be considered in the future for intraocular neoplasms.

### 3.3. In Vivo Sampling: Biopsy, Aqueocentesis and Vitreocentesis

As long as a tumor has not been diagnosed by histopathology, any diagnosis can only be a suspicion. Therefore, histopathological examination of the neoplasm, a sample of it, lymph nodes and metastases is an essential diagnostic step. In vivo sampling is an option if the eye is still clinically free of concurrent disease resulting from the neoplasia, and there seems to be a chance to preserve or restore vision, even when the potential systemic relevance of the intraocular neoplasia needs to be investigated [44]. For example, it may be possible to treat benign and still small medulloepitheliomas with selective curative therapy options (see Section 5 and Section 6). Aqueocentesis and vitreocentesis are routine procedures in equine ophthalmology, especially in diagnosing recurrent uveitis [24,81]. With only minor complications reported in cases of other indications (e.g., diagnosis of recurrent uveitis, treatment of ocular setariasis), they can be safely performed in the standing sedated horse under local anesthesia [81,82,83]. Additionally, it seems possible to sample the neoplasm directly, collect cells and submit them for histopathological examination [84]. Therefore, numerous techniques for intraocular tumor biopsy or fine-needle aspiration biopsy are well studied in human medicine and show reliable diagnostic value [85,86,87,88,89]. The potential of these procedures is unknown and needs to be further studied in the horse, particularly regarding advanced therapy options that may become available in the future. In dogs, their diagnostic value was studied, showing various successes of these procedures that may be dependent on surgical technique, tumor size and location [90]. The molecular and genetic characteristics of the individual tumor may be the foundation for targeted therapy, as there are studies on human intraocular tumors regarding oncolytic viruses and immunotherapy [91]. Possible complications of these procedures (e.g., bleeding, infection) must be taken into consideration and need to be further studied in cases of intraocular neoplasia in horses. However, if diagnostic–therapeutic consequences are not expected from in vivo sampling and if the bulbus and/or vision cannot be preserved due to the biological behavior of the tumor, the indication is questionable, and the procedure does not need to be performed on the animal.

### 3.4. Oncologic Work-Up and Tumor Staging

The diagnosis of internal neoplasia in horses is challenging [7,8,9,47,92], and until now, no standardized approaches in cases of intraocular neoplasms exist, despite single reports [44]. The existing procedures in equine oncology need to become a standard requirement while investigating intraocular neoplasia, especially the use of potential biomarkers that have been gaining in importance in equine oncology throughout the last few years [7,47,93,94,95]. Only routine use can result in reliable statements concerning their diagnostic value for investigating the systemic oncologic relevance of intraocular tumors in horses.

#### 3.4.1. Diagnostic Approach to Internal Neoplasia

Clinical examination: This remains the first step for every patient in equine practice and includes the medical history, as mentioned above (Section 3.1). Signs for internal neoplasia are regularly nonspecific and can include weight loss or edema [7,11,47,96]. In cases of intraocular neoplasia, the investigation of pain, including visual and neurologic behavior, seems to be important. Clinical examination of the lymph nodes plays a particularly important role in tumor staging. In horses, the submandibular lymph nodes are directly accessible by palpation and need to be routinely examined as part of the clinical exam. Initial studies provide references for ultrasonographic examination of clinically relevant lymph nodes that cannot be palpated [73].

Analysis of peripheral blood: Cancer can result in chronic inflammation or functional impairment of organs which can be measured with routine hematology and biochemistry [7,11,47]. Additionally, acute-phase proteins and protein electrophoresis should be added. These are generally unspecific markers, but are helpful when considering clinical effects, chronicity and prognosis [11,47].

Tumor biomarkers: As horses’ body cavities are not commonly accessible for detailed (sectional) imaging, biomarkers are gaining importance in equine oncology [47]. Studies in horses are limited, but preliminary studies report promising diagnostic value, especially for thymidine kinase 1, which was already studied as a proliferation marker in case of lymphoma in horses [47,93,94,95]. Micro RNAs are studied in human medicine as biomarkers of a prognostic and predictive nature for several diseases [7,32]. For the equine sarcoid [97,98], the first studies are investigating them, but their relevance for the diagnosis of internal neoplasia in horses is still unknown, despite potentially being promising. Other markers for internal neoplasia (e.g., hormones: erythropoietin, norepinephrine, normetanephrine; enzymes: alkaline phosphatase; antibodies/immunoglobulins: autologous antibodies, monoclonal gammopathy) are the subject of current research [47] and their relevance in cases of intraocular tumors needs to be assessed in the future.

Diagnostic imaging: Although imaging techniques are continuously improving in equine medicine, significant limitations result from the physical dimensions of horses’ body cavities, which are not commonly accessible for sectional imaging techniques [7,8,11]. However, a combination of all the results of the available and indicated imaging techniques (e.g., radiography, ultrasonography, endoscopy, computed tomography, magnetic resonance imaging, laparoscopy, thoracoscopy) often already allows for reliable diagnostic considerations [7,11].

Tissue sampling: Once a neoplasia is suspected and a working diagnosis is made, it needs to be confirmed by collecting neoplastic cells and submitting them for histopathological or cytological examination [7,11,20,30,87,89,90,99]. Regarding tumor staging, in addition to examining all clinically relevant regional and distant lymph nodes, sampling of regional lymph nodes should also be included in oncological diagnostics. This has also been reported in individual case reports for intraocular neoplasms [44]. Sampling of deeper lymph nodes, which may be performed with ultrasound guidance, requires further clinical investigation in horses, whereas ultrasound access to examine lymph nodes has initially shown promising results [73].

#### 3.4.2. Staging of Internal Neoplasia

Although treatment options for horses with internal and intraocular neoplasia may be limited, differentiated staging and grading of the neoplasia is essential for a reliable oncologic perspective and prognosis for the patient. The TNM (tumor, node, metastasis) classification system is routinely used in human oncology and can be transferred to equine oncology [7,96]. Especially in cases of intraocular tumors, standardized protocols to assess regional and distant lymph nodes and the search for possible metastases is underestimated, because there is little evidence-based knowledge about primary intraocular tumors. Although they are common in small animal medicine [7,96], staging systems for specific tumors in horses are lacking and further studies are required. As mentioned above, imaging modalities allow for a reliable examination of the tumor itself. Clinically relevant regional and distant lymph nodes are accessible by ultrasound, and references for the healthy lymph node were provided by Gremillet et al. [73]. Further studies need to report their clinical changes in cases of intraocular neoplasia, which emphasizes that the examination of lymph nodes needs to become part of the routine examination in cases of intraocular neoplasia. The assessment of metastases can be challenging in horses because of the well-known limitations of the imaging techniques in horses [11,69,71,100]. The two-dimensional black-and-white visualization, the penetration power using X-ray and the relatively low depth of penetration via ultrasonography do not allow for medical imaging of the large body cavities in depth or detail [77,100]. Computed tomography and magnetic resonance imaging are not commonly available to assess the equine thorax or abdomen due to size restrictions [77]. Diagnostic laparotomy or laparoscopy are gaining importance in these cases but still do not allow for complete examination [101,102]. In particular, direct imaging of the thoracic cavity is limited in the horse [103]. Little is known about the metastasis of primary intraocular tumors, and there are only suspected cases reported [44,46,48,57].

## 4. Clinicopathologic Classification of Intraocular Tumors in Horses

In human oncology, a continuous histopathologic and immunophenotypic classification system of tumors is provided by the World Health Organization including a particular system for tumors of the eye [1]. There have been attempts to classify equine neoplasia (e.g. lymphoma) in a comparable way [2]. As intraocular tumors in horses are only reported as single case reports or small retrospective studies, a comparable classification seems challenging at the moment, but we aimed to provide a structural overview as support for clinical work (Table 2). For a better understanding, the embryologic development of the eye and its functional structures is important to consider. The distinctive three-layer structure of the globe, consisting of a fibrous tunic (cornea, sclera), a vascular tunic (iris, ciliary body, choroid) and a nervous tunic (retina, optic nerve) and the embryological origin of the inner layers from neuro- (nervous tunic) and mesoectoderm (vascular tunic), makes it easy to classify tumors based on their tissue origin [104]. For example, in the case of neuroectodermal tumors, the possibility of expansion to the optic nerve and even intracranial invasion [25,26,27,38] is logical according to this classification. Furthermore, the tendency of mesoectodermal/uveal tumors to metastasize may be more likely than in neuroectodermal tumors due to their more consistent lymphatic and blood vessel supply [48,104]. Observing these basic principles allows for clinically relevant classification (Table 2) and facilitates the predictability of tumor behavior, enabling differentiated therapeutic considerations.

### 4.1. Neuroepithelial/Neuroectodermal Intraocular Tumors

The retina is of neuroectodermal origin, and the neuroectodermal intraocular tumors are commonly divided into those of the mature neuroepithelium and those deriving from the primitive medullary epithelium [23,52].

Medulloepithelioma and retinoblastoma derive from the primitive medullary epithelium and are summarized under the terms “primitive neuroectodermal tumor (PNET)” or “intraocular embryonal tumors”. The spontaneous occurrence of retinoblastoma in horses is controversially discussed [52], as the differentiation between medulloepithelioma and retinoblastoma, when deriving from the posterior retina, can be challenging [26,52,105].

The medulloepithelioma (Figure 1b, Figure 2b and Figure 7) is one of the most common intraocular neoplasia in horses and derives regularly from the ciliary body region [23,26,52,105]. Tissue formation deriving from the optic nerve [25,106] or infiltrating besides the posterior segment [54], the anterior segment (Figure 2b) [27,51,53,56] or the extrabulbar space [26,27,106] have been reported as well.

**Table 2:** Clinicopathological classification of reported intraocular tumors in horses *.

	CLINICOPATHOLOGICAL CLASSIFICATION	LITERATURE
neuroepithelial tumors	primitive medullary/neuroectodermal neoplasms	
	retinoblastoma	[52]
	medulloepithelioma, neuroectodermal	
	teratoid	[25,26,28,51,53,106]
	non-teratoid	[27,54]
	neoplasms of mature neuroepithelium	
	optic disc astrocytoma	[61]
	not specified/undifferentiated	[38,46]
uveal tumors	melanocytic uveal neoplasia	
	malignant melanoma	[17,29,48,58,60]
	benign melanocytoma	[48,59,60]
lymphatictumors	lymphoma	
	uveal T-cell lymphoma **	[44]
neoplasia metastatic to the eye	thyroid carcinoma	[41]
	melanoma (malignant) **	[48,49,107]
	adenocarcinoma	[43]
	lymphoma	[42]
	renal carcinoma	[108]

* may be incomplete; ** may be only suspected.

Especially in cases when the medulloepithelioma is not arising from the ciliary body or even from the region of the optic disc or nerve, clinical differentiation from retinoblastoma is challenging [26,51,52,53,105]. Histopathologic differentiation is possible, as medulloepitheliomas show larger and thicker rosette and tube structures with complex cell lining and a prominent lumen [26,105]. Non-teratoid medulloepitheliomas [25,27,54,56] show only a proliferation of the medullary epithelium, whereas teratoid medulloepitheliomas [26,28,51,53] additionally contain heterotropic elements such as cartilage cells, neurologic cells and myoblasts. [52,53,105]. Further, teratoid medulloepitheliomas are reported with malignant [25,28,51] and benign [53] behavior in the horse. Although it is a pediatric neoplasm in humans, most of the cases are reported in the adult horse [23,25,53,54,56,105]

One case of retinoblastoma [52] has been reported in the horse (Table 2). Histopathologically, this was characterized by primitive neuroectodermal cells that were present as sheets, nodules and cords that formed rosette structures partly with a central lumen [52]. Necrosis, calcification and invasion of iris tissue and subretinal expansion were present [52].

Neoplasia of the mature neuroepithelium [38,46,61] is reported only sporadically and as mostly undifferentiated [38,46] in horses (Table 2). An optic disc astrocytoma [61] was reported without clinical signs of ocular pain or inflammation and without impairment of vision. The astrocytoma circumscribed the dorsomedial optic disc with absent retinal blood vessels dorsal of it [61]. Another neuroepithelial tumor, which was surgically removed, was described in a mare concurrent with a metastatic mammary carcinoma [46]. In another case, an intraocular neuroepithelial tumor showed spreading into the retrobulbar space and calvarium [38]. It showed mixed neoplastic cells of the primitive neuroepithelium [38].

Due to the diagnosis usually occurring in late stages of the disease, prognosis regarding the preservation of vision and the globe is regarded as poor. Regularly, enucleation of the affected eye is performed when the neoplasia causes concurrent ophthalmic or peribulbar disease [25,26,27,28,38,51,52,53,54,56,106]. Better prognosis in cases of neuroepithelial tumors arises in human medicine as a result of early detection of the tumor and may be translatable to equine medicine too [109,110].

### 4.2. Uveal Intraocular Tumors: Melanocytic Neoplasia

Melanocytic neoplasia is described as a common intraocular neoplasia in horses (Table 2) [23,48,105]. It derives from the anterior uvea (iris, ciliary body) that develops out of the embryologic mesoectoderm [48,49,60,105]. Usually, it derives from the iris into the anterior chamber (Figure 2a and Figure 5a) [57,59,60,111], but can also affect and infiltrate the iridocorneal angle or the posterior uvea (ciliary body, choroidea) [48,55,60,112]. Epibulbar melanoma has been reported as well [17]. Gray horses are overrepresented, but it is also reported in cremellos and horses of other colors [48]. The presenting complaint was the recognition of an intraocular mass in most of the cases, with or without concurrent ophthalmic disease [48]. Single intraocular melanocytic neoplasia are reported, as well as cases with melanoma in other parts of the body [40,48,49,107,113].

Clinically, it is important to assess the patient for concurrent disease, such as keratitis, glaucoma, cataract or uveitis [24,29,50] (Table 1), and to assess the precise dimensions of the tumor using clinical examination and the described imaging techniques, in particular ultrasonography, as described above [50,64,67].

Intraocular melanocytic neoplasia is reported as benign melanocytoma [48,59,60] or malignant melanoma [29,48,58,60]. The histopathologic nature of melanocytic neoplasia is well described [107,113] and can vary in intraocular appearance concerning location, tissue infiltration, predominant cell type, mitotic count and pigmentation [48,105].

The most common therapeutic approach is surgical removal via sector iridectomy in cases where the anterior iris is affected, and this has good prognosis for the preservation of vision in early stages of the disease [59,111]. Enucleation is an option in cases of a posterior location or concurrent ophthalmic disease [5,48,59,60,114].

### 4.3. Intraocular Lymphoma

One single case of a suspected primary intraocular T-cell lymphoma has been reported [44]. It was diagnosed in vivo by aqueocentesis and cytologic examination. Additionally, a systemic oncologic work-up was presented by the authors [44]. Cytologic examination of the aqueous humor revealed a large amount of lymphoid cells that dominated and were mixed with disintegrated cells and cellular debris [44]. Based on the lymphoid cell population, a diagnosis of lymphoma was made [44]. During post-mortem examination, the lymphoid cells were found infiltrating large parts of the uvea and were identified as T-lymphocytes by immunohistochemistry [44].

This single case shows the rarity of primary intraocular lymphoma. However, this proves that it must be considered a differential diagnosis for diseases of the inner eye. Not least, this requires clearly differentiating the ocular manifestations of systemic lymphoma [2,39,40] from the primary origin of the disease (Table 2).

### 4.4. Metastasis from Systemic Neoplasia

Besides the well-studied ocular manifestations of lymphoma in horses [42], only single case reports exist reporting metastasis of systemic neoplasia to the eye [41,43,48,49,107,108].

In these cases, a reflected analysis of oncological assessment is essential in order to evaluate the patient adequately (Section 3) and needs to become routinely performed in cases of ocular neoplasia in horses [7,96]. This will allow for further investigations concerning relevant metastases from systemic neoplasia to the inner eye.

## 5. Therapy: Interdisciplinary and Interspecies Overview and Outlook

Surgical removal via enucleation or exenteration of the affected eye and adnexa is the most common therapeutic choice for intraocular neuroepithelial neoplasia [25,26,27,28,38,51,52,53,54,56,106]. However, recurrence and infiltration into surrounding tissue have been reported, even after enucleation has been performed [26,27,38,51]. In addition, the increased expectations of horse owners regarding treatment options that allow for the preservation of vision make it necessary to consider advanced treatment strategies.

In human medicine, following an interdisciplinary oncologic work-up, the development of a multimodal therapeutic approach is routinely performed [3,4,5,114]. This approach is also gaining importance in small animal medicine [115,116,117] and should also be introduced in equine medicine. It can be assumed that a comparable variety of applications for clinical use in horses with intraocular tumors is still a long way off. However, taking into account the currently proven and promising approaches, the idea of a multimodal therapeutic approach should also be the standard in equine oncology.

### 5.1. Surgery

**Figure 8:**
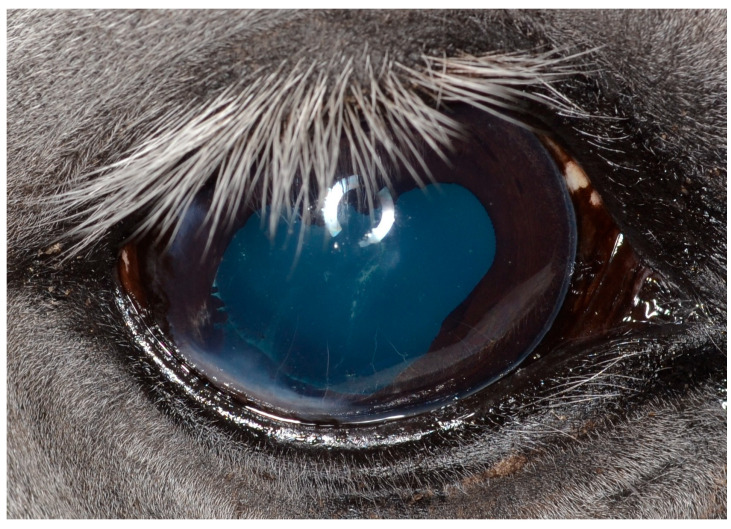
Clinical case with iris melanoma after sector iridectomy at 6 weeks after surgery.

Surgical removal of iris melanoma using sector iridectomy (Figure 8) is common practice in equine ophthalmic surgery [59,111,118,119]: The anterior chamber is accessed through a limbal/corneal incision that allows for adequate exposure to the affected iris tissue. The anterior chamber is stabilized, and the iris tissue is carefully retracted from the anterior lens surface in the incision. The iris tissue is incised by scissors or cautery. The corneal incision is closed, and the viscoelastic agent is removed by irrigation/aspiration from the anterior chamber, as described elsewhere [118,119,120].

Postoperative care includes the control of iridocyclitis, and a risk of developing postoperative glaucoma exists [119]. The outcome is quite good for isolated iris melanoma [59,111]; however, prognosis is guarded when the neoplasm extends to the basal iris and ciliary body [119,120].

Future considerations should include the recreation of a functional pupil by pupilloplasty, which is performed in human ophthalmic surgery [5,121], to reconstruct a functional pupil, as this is the main functional impairment resulting from sector iridectomy alone. The pupillary margin can be sutured intraocularly [121]. Although this will still lead to anisocoria, it allows normal pupil function to be maintained [119,121]. In cases of a more complex structural appearance of the tumor, an adjunctive or sole treatment with diode laser photocoagulation should be considered, which is used commonly in small animal practice [112].

**Figure 9:**
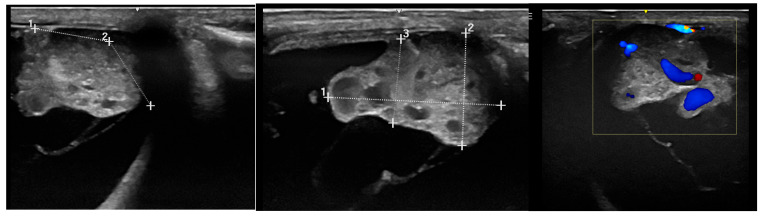
Investigation of intraocular medulloepithelioma by diagnostic ultrasound an example of presurgical considerations. Planning of surgical approach (**left**), investigating tumor dimensions (**middle**) and vascularization by Doppler (**right**).

The surgical approach to neoplasia of the posterior segment is described in human medicine (iridocyclectomy, sclerouvectomy) [3,4,5,114] and small animal ophthalmic surgery (iridocyclectomy) [119,122]. Currently, there is no research describing these procedures for equine intraocular neoplasia. Their potential success, particularly regarding vision and possible recurrence, appears highly questionable at this point in time due to the structural dimensions and functional characteristics of the equine eye. The most frequent complications are retinal detachment, intraocular hemorrhage, incomplete tumor removal or cataracts in human [5] and small animal medicine [122], and it can be assumed that these risks are significantly higher in horses due to the size of the globe and, in particular, the vitreal cavity [22,104]. Detailed preoperative planning, in particular using medical imaging (Figure 9), and even collaboration with human ophthalmic surgeons may make these surgical procedures accessible for the horse.

Diode lasers for veterinary ophthalmology are commercially available. In dogs and cats, their use for intraocular neoplasia is described for the transcorneal photocoagulation of presumed melanocytic tumor or the ablation of iris hyperpigmentation in cats [112,123]. In horses, diode lasers are used for the transcorneal reduction of iris cysts, but their limited penetration power may restrict their usability in cases of solid iris melanoma in horses [48]. The adjuvant use of laser therapy, especially in cases where surgery is difficult, seems promising. In human medicine, the diode laser is used for small retinoblastomas, in addition to its use in melanocytic intraocular neoplasia [4].

As part of surgical tumor therapy, it is also necessary to consider the removal of affected lymph nodes. There are no case reports of this being performed after positive cytological findings in horses with intraocular tumors. In case of penile squamous cell carcinoma in horses, sampling of the relevant lymph nodes is standard practice, and removal in cases of positive cytology improves the prognostic expectations [124,125].

### 5.2. Chemotherapy

Cytotoxic drugs target rapidly dividing cells and are commonly used for cancer therapy in human medicine; they are gaining importance in small companion animals [116,126,127,128,129,130]. In equine oncology, their use for internal neoplasia actually is limited to treatment of lymphoma, and even for this disease only small retrospective case series exist [131]. In cases of external neoplasia (sarcoid, squamous cell carcinoma), their local use is common practice as the sole or adjuvant procedure [126,127]. For intraocular tumors, chemotherapy is not described in veterinary medicine. In human medicine, chemotherapy is predominantly used for binocular tumors, as an adjuvant therapy in large neoplasia or metastatic neoplasia and as a sole treatment approach in intraocular tumors with sub-retinal effusion or retinal detachment [4]. Human oncology may be the basis of considerations for its clinical use in veterinary medicine. However, the low number of cases in horses, the financial and time investment for the horse, the yet unforeseeable side effects and complications, and the special pathological and anatomical characteristics of the equine eye mean that the clinical applicability of chemotherapy in horses in these cases is still a long way off.

Table 3 gives an overview of the classes of chemotherapeutics and selected substances used for intraocular neoplasia in humans, which serves as a possible consideration basis for use in horses. Their systemic or local administration, for example through retrobulbar or local intra-arterial or intratumoral injections [4], may be possible in horses as well. Alkylating agents such as cisplatin or carboplatin bind to DNA, change its structure and can interfere with DNA transcription, replication and repair mechanisms [126]. They are used to treat intraocular tumors in humans through systemic intravenous administration [3,4], and protocols for their local administration in cutaneous neoplasia are well proven for the horse [126,127]. This may be a resource of basic knowledge to develop protocols for local or systemic administration to treat intraocular neoplasia in the horse. The antitubulin agent vincristine blocks intracellular tubulin formation and interrupts cellular mitosis in the metaphase [126]. It is part of the treatment protocols systemically administered for equine lymphoma [131]. In combination with etoposide and carboplatin, it is used for systemic or local intravascular chemotherapy in human medicine [3,4]. These substances or their combinations may be an option for promoting the regression of intraocular tumors in horses in the future.

Cutaneous neoplasms, including periocular sarcoids and squamous cell carcinoma, are successfully treated using electrochemotherapy [130,132,133]. As a single or adjuvant treatment to surgery, cisplatin or bleomycin are commonly administered into the tumor, followed by electrical pulses [133]. These lead to cell wall instability and are intended to increase the uptake of chemotherapeutic agents into the tumor cell [130,132,133]. For use in intraocular neoplasms, the effect of electric shocks on the inner eye must be taken into account, especially when transferring the protocols for cutaneous neoplasms: human medicine reports inflammation of the inner eye [134] and cataract formation as a result of electrical injury [134,135,136] in numerous cases. Nevertheless, the potential use of the electrochemotherapeutic principle is studied in human medicine for intraocular tumors, especially uveal melanoma [129]. Therefore, it may be possible to develop protocols for the electrochemotherapeutic treatment of intraocular neoplasms in horses. Due to the occurrence of intraocular melanoma in horses, horses may be used as a model for human oncologic studies, which is discussed below.

Adverse effects in horses are rarely reported and mostly characterized as mild and manageable [126,127,131]. Specific studies concerning adverse effects in horses are lacking, but the first studies in small animals are available [117]. For their use for intraocular tumors in humans, possible adverse and side effects are described including a loss of vision and an eyeball [4].

### 5.3. Radiation Therapy

For intraocular tumors in animals, radiation therapy has not been reported so far. In horses, external beam radiation therapy or brachytherapy is used for cutaneous, mainly periocular [137,138,139] tumors. In one case series, radiation therapy was used alone or in combination with debulking surgery for the treatment of non-cutaneous neoplasia of the head (fibroma, fibromasarcoma, lymphoma, carcinoma, osteosarcoma, ameloblastoma and myxosarcoma), with promising therapeutic success and manageable adverse effects [140]. In small animals, radiation therapy has a much longer history, and more experience has been gained. It is reported in the treatment of many localized infiltrating tumors, in which wide surgical margins cannot be achieved. It can be used as monotherapy or in combination with other treatment modalities including chemotherapy, surgery and immunotherapy. One of the typical indications is the treatment of brain tumors, either alone or in combination with surgery, with good therapeutic success so far [141,142,143,144].

Human oncology uses proton beam radiation and plaque brachytherapy for the treatment of uveal melanoma or choroid metastatic carcinoma [4]. For the former, surgical placement of marker rings for the guidance of radiation therapy is necessary [4]. It is emphasized that radiation therapy can be used alone or as an adjuvant therapy within a multimodal therapeutic approach [3,4], and this has already been considered in veterinary medicine [128,137,138,139,140,145].

The outlook of human medicine seems to be promising, but future studies need to evaluate the therapeutic value of radiation therapy for intraocular neoplasia in horses. However, the availability of proton radiation facilities for horses seems unrealistic at the moment. Nevertheless, radiotherapy of affected lymph nodes may offer a potential adjuvant treatment, not only in cases of intraocular neoplasia.

Several challenges occur when using radiation therapy in veterinary and equine oncology [128,137,140,145]. External beam radiation therapy is available in veterinary medicine, where electrons are delivered to treat superficial neoplasia and photons are used for deeper neoplasia [137,139]. Cross-sectional imaging advancements help to increase the accuracy of beam orientation, machine settings and optimal radiation doses and protocols [137,139,141]. The accurate, repeatable positioning of the patient on the basis of sectional imaging is still challenging in veterinary medicine and will be a future challenge in horses as well due to their physical dimensions [137]. General anesthesia is commonly needed to immobilize the patient for proper positioning [137,138].

When radiotherapy was first used in horses, adverse and side effects of radiation therapy occurred frequently [137,140], especially those affecting sensitive structures of the eye (keratopathy) [146,147]. Adequate planning, precise and accurate positioning of the patient and the use of advanced treatment modulating techniques (intensity-modulated radiation therapy (IMRT), volumetric modulated arc technique (VMAT) with on-board imaging) have significantly reduced the occurrence of the described problems [147].

## 6. Comparative Oncology: Perspective Considerations

One in three people develop cancer in their lifetime, which makes oncology one of the most relevant fields in human medicine research [6]. Companion animals are used as models to evaluate cancer biology, as well as diagnostic or therapeutic options before their translation to clinical use in humans [6,148,149]. Animal models still remain the best option to investigate cancer’s disease onset, progression and metastasis [150]. Since intraocular melanoma and medulloepithelioma are the most common types of intraocular neoplasia in horses, it is worth considering whether they may serve as a model for human cancer research.

Uveal melanoma accounts for about five percent of all primary human melanoma cases, but is the most common intraocular malignancy in adults [3,5,114]. Because of its malignancy, it remains a clinical challenge in humans. Mortality rates between 30 and 40 percent as a result of predominantly liver and colorectal metastases are reported [114], making it one of the most lethal cancers [150]. Therefore, currently, different laboratory animal models are used; for example, genetically modified mice that spontaneously develop (cutaneous) melanoma and the engrafting of tumor cell lines including the inoculation of tumor cell lines and xenografts in laboratory animals are used [150,151]. Thus, animal models serve to answer very specific questions, but none of them meet the molecular genetic characteristics of human uveal melanoma, which is why further preclinical models are constantly being researched [150].

Since the biological and molecular–genetic characteristics of uveal melanoma differ greatly between humans and animals, there is currently no animal model with spontaneous occurring uveal melanoma for human cancer research [150,151,152,153,154,155]. Rats, cattle, cats, chickens and dogs have been studied, but there are no investigations on horses [150].

It seems unlikely that the horse could be used as an animal model for molecular genetic research on human uveal melanoma, but similarities in biological behavior may be helpful, particularly in the investigation of therapeutic options. The biological behavior of canine uveal melanoma (30% malignant, 5% metastatic) [156], as well as equine uveal melanoma [48,60,113,156], is well understood, whereas the ratio of malignant to benign tumors and the metastatic potential of equine uveal melanoma remains unknown [48]. Concerning biological behavior, the canine model seems to be promising [150,151,157], while the horse may be a model for the assessment of therapeutic options before translation to humans. For example, current laboratory studies present promising results of electrochemotherapy with bleomycin in cases of primary and metastatic uveal [129,158] and conjunctival [159] melanoma models in vitro. The horse may be a sufficient model for in vivo investigation. The canine model should also be taken into consideration, especially concerning the challenges in metastatic disease, which remains the major clinical challenge in humans [5,114,157,160,161].

Human ciliary body medulloepithelioma is a rare condition that occurs regularly in children [109,110], although it has been reported in adults as well [162]. In contrast to the horse, retinoblastoma is the most common primary intraocular tumor in humans that is diagnosed regularly before the age of five years [3]. In veterinary medicine, intraocular medulloepithelioma is regularly treated by enucleation of the affected eye in horses [26,27,28,38,51,56,106], as well as in small animals [163,164,165]. Concerning these intraocular epithelial tumors, the potential of comparative oncologic considerations is still unknown but treatment options from human medicine may be translated to veterinary medicine. As described above (Section 5.1), advanced surgical therapy (e.g., iridocyclectomy, sclerouvectomy) [3,4,5,114,166] may be translated to veterinary medicine. However, this translation from human medicine should also lead to the conclusion that surgical therapy can only be part of a multimodal approach and must be supplemented by radiotherapy or chemotherapy [109,110,167,168,169,170]. This requires further research into the molecular and genetic characteristics of intraocular medulloepitheliomas in animals, so that findings from human research can perhaps be transferred or specific targeted therapy options (e.g., chemotherapy, immunotherapy) based on human medical research can be developed [109,110,168,169,170,171,172,173].

## 7. Final Considerations and Conclusions

Intraocular neoplasms in horses have a low incidence but can lead to variable clinical signs and require differentiated diagnostics. A systemic oncological work-up must become routine in equine ophthalmology. In particular, comprehensive tumor staging is fundamental in cases of intraocular neoplasia.

Diagnostic options have advanced significantly in recent years. Further studies are required to validate the use of these techniques for the examination of intraocular tissue formations. Overall, reliable diagnostic options are available, particularly regarding the planning of therapeutic interventions. The clinicopathological classification of tumors based on their tissue origin allows for an assessment of tumor behavior for prognostic evaluation and consideration of therapeutic approaches.

Surgical treatment of anterior melanocytic neoplasms of the iris is successful in horses, but surgical options for other neoplasia are missing. Human medicine provides examples of multimodal therapeutic approaches for cases of intraocular neoplasms, the basic principles of which may be transferable to equine ophthalmology. While radiotherapy for intraocular neoplasms is unlikely to be available for horses in the foreseeable future, chemotherapeutic protocols appear feasible for clinical trials. On the other hand, horses and small animals may serve as a model for human medicine research.

In conclusion, this review demonstrates that differentiated diagnostics and staging, a fundamental oncological understanding and the development of individualized multimodal therapeutic concepts may improve the prognosis for horses with intraocular tumors in the future.

## Figures and Tables

**Table 1:** Parts of an ophthalmic examination [50] and exemplary findings that can be a result of intraocular neoplasia.

Diagnostic Step	Possible Findings	Literature
evaluation for symmetry from front of head	exophthalmos, buphthalmos, strabismus	[13,17,26,27,38,51]
	deformation of the eyeball	[17,27]
vision testing	impairment of vision, blindness, neurologic/neuroophthalmic deficits	[13,25,26,27,28,48,51,52,53,54,55]
palpebral and pupillary light reflexes		
overview examination by direct illumination	ocular discharge, epiphora	[46]
	conjunctival/scleral hyperemia, chemosis	[25,27,52,53,56]
	corneal edema, opacification and neovascularization, endothelial opacities	[26,29,43,44,48,51,53,57,58,59]
	anisocoria (miosis, mydriasis), dyscoria	[44,48,59]
tonometry	hypertone/glaucoma	[26,27,29,48,53]
detailed examination of anterior segment (transillumination and biomicroscopy)	tissue formation (anterior segment)	[26,29,38,43,48,52,53,56,57,58,59,60]
	flare, hyphema, hypopyon, fibrin	[22,43,44,56]
	iris: rubeosis iridis, thickening, pigment anomalies, tissue formation	[43,44,46,56,57,59]
inducing mydriasis	delayed or not possible	[44,52]
detailed examination of posterior segment (transillumination, biomicroscopy, retro-illumination, direct and indirect ophthalmoscopy)	tissue formation (posterior segment)	[25,28,38,52,54,55,61]
	vitreal hemorrhage, fibrin, flare, degeneration, syneresis	[25,26,52]
	lens: opacification, subluxation, luxation, synechiae	[29,48,52,57]
	abnormal neuroepithelium and choroidea (retinal detachment, tissue formation, chorioretinal lesions, …)	[25,43]

**Table 3:** Cytotoxic drugs (examples used in horse) [126,127,131], their use for intraocular tumors in humans [3,4] and their potential for clinical use in horses with intraocular neoplasia (marked green).

Group	Substances	Used in Human Intraocular Neoplasia [3,4]	Use in Horses [126,127,131]
alkylating agents	cyclophosphamide		lymphoma
	chlorambucil		squamous cell carcinoma
	melphalan		
	lomustine		squamous cell carcinoma
	cisplatin		sarcoid, sarcoma, melanoma, hemangiosarcoma
	carboplatin		sarcoid, sarcoma, melanoma, hemangiosarcoma
antitumor antibiotics	doxorubicin		lymphoma, sarcoid, melanoma, carcinoma
	mitomycine C		lymphoma, hemangiosarcoma, squamous cell carcinoma, melanoma
	bleomycin		sarcoid
	etoposide		
	topotecan		
antitubulin agents	vincristine		lymphoma
corticosteroids	prednisolone		lymphoma, mast cell tumor
	dexamethasone		lymphoma, mast cell tumor
antimetabolites	cytosine arabinoside		lymphoma
	5-fluorouracil		sarcoids, squamous cell carcinoma
miscellaneous	l-asparaginase		lymphoma

reported in horses OR humans; reported in both, humans and horses.

## Data Availability

No new data were created or analyzed in this study.
